# Refining immunotherapeutic approaches to high‐risk neuroblastoma based on tumor genomic profiles

**DOI:** 10.1002/1878-0261.12880

**Published:** 2020-12-26

**Authors:** Liron D. Grossmann, John M. Maris

**Affiliations:** ^1^ Department of Pediatrics and Center for Childhood Cancer Research The Children’s Hospital of Philadelphia Perelman School of Medicine at the University of Pennsylvania Philadelphia PA USA

**Keywords:** 11q deletion, anti‐GD2 therapy, immune checkpoint inhibition, microRNAs

## Abstract

In this issue, Coronado *et al*. attempt to improve our understanding of the factors affecting the response to immunotherapy in a large subset of high‐risk neuroblastoma with hemizygous deletion of chromosome 11q. By using several computational approaches, the authors study potential transcriptional and post‐transcriptional pathways that may affect the response to immunotherapy and further be leveraged therapeutically in a biomarker‐directed fashion.

## Graphical abstract

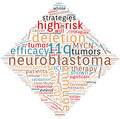

**Abbreviations**
ADCC: antibody‐dependent cellular toxicity
ICI: immune checkpoint inhibition
IDO1: indoleamine 2,3‐deoxygenase 1
IL‐10: interleukin‐10
PD‐L1: program death ligand 1
SCAs: segmental chromosomal alterations
TGF‐beta: tumor growth factor beta
TME: tumor microenvironment

While immune checkpoint inhibition (ICI) strategies have shown significant activity in subsets of adult malignancies, the pediatric clinical experience with these agents has largely been disappointing [1, 2]. This is perhaps not too surprising due to low mutation burden and general lack of an inflamed tumor microenvironment (TME) for many childhood cancers. However, chimeric antigen receptor‐engineered T cells have shown spectacular efficacy in refractory acute lymphoblastic leukemia [3], and a variety of monoclonal antibodies targeting the disialoganglioside GD2 that induce antibody‐dependent cellular cytotoxicity (ADCC) have shown efficacy in high‐risk neuroblastoma [4, 5, 6]. Both of these strategies target lineage‐restricted cell surface molecules, and it is currently unknown if efficacy can be enhanced in combination with ICI or other immunotherapeutic strategies.

Despite the efficacy of GD2‐targeting immunotherapies in high‐risk neuroblastoma, a pediatric cancer arising from the developing sympathetic nervous system, survival rates remain mired at ~ 50% despite highly intensive chemoradiotherapy that typically precedes anti‐GD2 therapy [7]. Of note, the therapy shows significant on‐target off‐tumor toxicity as GD2 is expressed on pain fibers. While many investigators are working on strategies for further improving neuroblastoma outcomes by combining anti‐GD2 monoclonal antibodies with chemotherapy, including incorporating into frontline induction chemotherapy, many others are in search for alternative immunotherapeutic targets that may be more tumor‐specific.

High‐risk neuroblastoma is broadly subdivided into two major cohorts: younger patients (toddlers) whose tumors show *MYCN* amplification, 1p deletion, and *TERT* overexpression; and older patients whose tumors harbor 11q deletion, occasionally along with other recurrent segmental chromosomal alterations (SCAs), maintain chromosome ends via the alternative lengthening of telomere mechanism, and generally show a slightly more inflamed TME [8, 9, 10, 11]. It remains unknown if these or other genomic alterations are associated with anti‐GD2 efficacy or how they may coordinate immune escape. Importantly, it has been known since the 1980s that *MYCN* amplification is directly associated with low major histocompatibility complex expression [12], and it has been confirmed more recently that *MYCN*‐amplified neuroblastomas have one of the most immune cell excluded TMEs of all human cancer [13]. The 11q‐deleted group does show more of an immune infiltrate [11, 14], but whether this is sufficient to contribute to anti‐tumor immunity remains unknown.

In this issue, Coronado *et al*. begin to address these questions by focusing on the large subset of high‐risk neuroblastoma that show hemizygous deletion of chromosome 11q. Despite years of intensive research, the mechanism by which 11q loss contributes to tumorigenesis remains poorly understood (Mlakar *et al*)[15]. The loss of one copy of several candidate protein‐coding genes located on 11q has been proposed; however, no recurrent second hit of a putative tumor suppressor gene has been found to date. MicroRNAs (miRNAs) may play a role, with let‐7a‐2 that maps to 11q shown to directly suppress *MYCN* mRNA [16], perhaps explaining, at least in part, the significant anti‐correlation of 11q deletion and *MYCN* amplification in high‐risk neuroblastoma [8]. Here, Coronado *et al*. extend the published literature by further defining the mechanisms of immune suppression in patients with high‐risk neuroblastoma harboring 11q deletion, such as overexpression of program death ligand 1 (PD‐L1), interleukin‐10 (IL‐10), tumor growth factor beta (TGF‐beta), and indoleamine 2,3‐deoxygenase 1 (IDO1). These might be leveraged therapeutically in a biomarker‐directed fashion, by combining anti‐GD2 immunotherapy with ICI therapy in patient with 11q deletion.

The authors used a well‐described deconvolution method to describe the immune cellular composition in two published independent RNA sequencing neuroblastoma datasets used as discovery and validation cohorts. They found in both datasets that tumors with 11q deletion showed a higher proportion of CD8+ and resting CD4+ memory T cells, as well as of resting (M0) and polarized macrophages (M1 and M2) compared to neuroblastoma tumors without 11q deletion, the majority of which show *MYCN* amplification. There was some discordance regarding NK cells, with the proportion of activated NK cells being significantly lower in the 11q‐deleted cohort in the discovery dataset, but not in the validation cohort. As the efficacy of anti‐GD2‐mediated tumor killing largely depends on NK cells, which are the effectors of ADCC, it will be crucial to investigate in additional collaborative research whether the low numbers of activated NK cells are a hallmark of 11q‐deleted neuroblastoma tumors. Overall, these analyses confirm and extend prior analyses showing that older high‐risk neuroblastoma patients whose tumors harbor 11q deletion do have immune effectors cells in the TME, and these may be poised to contribute to an adaptive immune response.

While exploring the mechanisms potentially underlying these observations, the authors then show that known mediators of intra‐tumoral immune suppression such PD‐L1, IL‐10, TGF‐beta, and IDO1 are differentially overexpressed in cases with 11q deletion. They then recapitulate the well‐described fact that 11q deletions often occur with other SCAs [17]. While aneuploidy is associated with immune evasion and lack of efficacy of ICI in adult cancers with whole chromosome gains [18], the role of near diploidy with segmental chromosomal arm aberrations as seen in neuroblastoma is unclear and will be an important question for future studies. Additionally, the authors postulate that miRNAs may contribute to an immunosuppressive TME, an interesting finding that will require mechanistic validation in appropriate model systems.

Taken together, this work further highlights the importance of defining clinically relevant molecular subsets of human cancer to more precisely develop immunotherapeutic and other anti‐cancer strategies. The authors hypothesize that 11q deletion may be a biomarker for synergistic efficacy of anti‐GD2 immunotherapy with ICI therapy. Due to the rarity of high‐risk neuroblastoma, more intensive international cooperation will be necessary to both prove and extend this hypothesis through rigorous biomarker‐defined clinical trials.

## Conflict of interest

The authors declare no conflict of interest.

## Author contributions

Drs. Grossmann and Maris co‐wrote this commentary.
